# A bibliometric analysis of studies on the gut microbiota in cardiovascular disease from 2004 to 2022

**DOI:** 10.3389/fcimb.2022.1083995

**Published:** 2023-01-06

**Authors:** Ming Sheng, Shuquan Xu, Wei-wei Chen, Fa-quan Li, Yi-ming Zhong, Yi-xiang Ouyang, Yong-ling Liao, Ping Lai

**Affiliations:** ^1^ Department of Library, Gannan Medical University, Ganzhou, Jiangxi, China; ^2^ School of Basic Medicine, Gannan Medical University, Ganzhou, Jiangxi, China; ^3^ Department of Pharmacology, Gannan Medical University, Ganzhou, Jiangxi, China; ^4^ Department of Cardiology, The First Hospital of Gannan Medical University, Gannan Medical University, Ganzhou, Jiangxi, China; ^5^ Key Laboratory of Prevention and Treatment of Cardiovascular and Cerebrovascular Diseases, Ministry of Education, Gannan Medical University, Ganzhou, China

**Keywords:** gut microbiota, cardiovascular diseases, bibliometric analysis, VOSviewer, CiteSpace, WoS

## Abstract

**Background:**

Increasing evidence indicates that the gut microbiota (GM) is linked to cardiovascular disease (CVD). Many studies on the GM in CVD have been published in the last decade. However, bibliometric analysis in this field is still lacking.

**Methods:**

On 30 September 2022, a search of the Web of Science™ (WoS; Clarivate™, Philadelphia, PA, USA) yielded 1,500 articles and reviews on the GM and CVD. Microsoft Excel and CiteSpace and VOSviewer software were used to analyze publication trends and research hotspots in this field.

**Results:**

Our search generated 1,708 publications on the GM in CVD published between 2004 and 2022, and 1,500 articles and review papers were included in the final analysis. The number of publications relating to the GM in CVD increased from 1 in 2004 to 350 in 2021. China (485 publications, 9,728 non-self-citations, and an H-index of 47) and the USA (418 publications, 24,918 non-self-citations, and an H-index of 82) contributed 32.31%, and 27.85%, respectively, of the total number of publications. Examination of the number of publications (Np) and number of citations, excluding self-citations (Nc), of individual authors showed that Y. L. Tian (Np: 18, Nc: 262, and H-index: 12), from China, is the most productive author, followed by R. Knight (Np: 16, Nc: 3,036, and H-index: 15) and M. Nieuwdorp (Np: 16, Nc: 503, and H-index: 9). The Chinese Academy of Medical Sciences and Peking Union Medical College accounted for the largest number of publications (Np: 62, Nc: 3,727, and H-index: 13, average citation number (ACN): 60.11). The journal *Nutrients* had the most publications (Np: 73, Nc: 2,036, and ACN: 27.89). The emerging keywords in this field were “monooxygenase 3” (strength 3.24, 2020–2022), “short-chain fatty acid” (strength 4.63, 2021–2022), “fatty liver disease” (strength 3.18, 2021–2022), “metabolic disease” (strength 3.04, 2021–2022), “Mediterranean diet” (strength 2.95, 2021–2022), “prevention” (strength 2.77, 2021–2022), and “intestinal barrier” (strength 2.8, 2021–2022).

**Conclusion:**

Publications on the GM in CVD rapidly increased in the last decade. The USA was the most influential country in publications in this field, followed by China. The journal with the most publications was *Nutrients*. Monooxygenase-3, short-chain fatty acids, fatty liver disease, metabolic disease, the Mediterranean diet, intestinal barrier, and prevention are the current hotspots or potential hotspots for future study.

## Introduction

The gut microbiota (GM) comprises microorganisms, including bacteria, viruses, and fungi, which live in the digestive tract (Thursby and Juge, 2017; Chen et al., 2021). The total number of these microorganisms in the microbiota is more than 10 trillion (Witkowski et al., 2020; Ley, 2022). The primary role of the GM is to assist in the fermentation of non-digestible substrates (Valdes et al., 2018), but studies have indicated that the GM causes several diseases, including infectious diseases (Libertucci and Young, 2019), hypertension (Li et al., 2017), atherosclerosis (Ma and Li, 2018), and thrombus formation (Duttaroy, 2021). The GM releases endotoxins or metabolites that trigger the primary immune response, causing systemic inflammation (Rooks and Garrett, 2016; Brandsma et al., 2019). An appropriate balance of microorganisms in the gastrointestinal tract is necessary for the maintenance of several bodily processes, whereas an imbalance is detrimental to the host (Fujimura et al., 2010; Hasan and Yang, 2019). GM transplantation or modulation is a promising method for treating several diseases caused by GM dysbiosis (Liu et al., 2017; Settanni et al., 2021). Our knowledge of the GM has gradually increased over time (Trejo-Castro et al., 2022).

Cardiovascular disease (CVD) is one of the leading causes of death worldwide (Roth et al., 2020). The factors responsible for CVD are variable, and the mechanisms complicated. Studies in the last two decades have implicated the GM in CVD (Ordovas and Mooser, 2006). The GM has been found to be related to atherosclerosis, as bacterial DNA has been detected in atherosclerotic plaques (Koren et al., 2011). Many studies in recent decades have confirmed that the GM contributes to the pathophysiology of CVD, and understanding the underlying mechanisms could provide novel therapeutic strategies for treating CVD (He et al., 2015; Blandino et al., 2016; Li et al., 2017). Hypertension and dyslipidemia are two dominant risk factors for CVD and are related to the alteration of the GM (Li et al., 2017; Chi et al., 2019; Zwartjes et al., 2021). Metabolism-related diseases, such as type 2 diabetes and obesity, have over time become the main health burdens globally, and both of them are associated with the GM, a major factor implicated in the development of CVD (Piche et al., 2020). Further research is under way to explore the possible molecular mechanism of how the GM contributes to the incidence of CVD. Metabolites synthesized by the GM, such as choline trimethylamine/trimethylamine oxide (TMA/TMAO), promote the development of atherosclerosis/cardiorenal fibrosis by binding to amine-associated receptors (Chen et al., 2016). Lipopolysaccharide modulates vascular function *via* the toll-like receptors 2, 4, and 9 (Medvedev et al., 2000), and short-chain fatty acids negatively affect blood pressure via G-protein-coupled receptors 41 and 43 (Natarajan et al., 2016). More studies in this area are still under way.

Bibliometrics analysis based on VOSviewer or CiteSpace is widely used to systematically assess the achievements, hotspots, and research trends in a specific area based on the literature published in this field. Bibliometric indicators include cooperation between authors, countries, and institutions, as well as keywords and references (Rousseau, 2014). Bibliometric analysis summarizes the abundant data and findings in a specific field. In recent years, it has been widely applied in many fields of medicine, such as developing drugs for CVD (Lai et al., 2022), the role of exosomes in CVD (Ma et al., 2021), the role of signaling pathways in the development of diseases (Shi et al., 2020), and cardiac regeneration (Ma et al., 2021). Over the last two decades, more than 1,500 pieces of literature on the GM in CVD have been published. However, bibliometric analysis in this field is still lacking.

The current study summarized the most recent progress, evolutionary path, study hotspots, and potential research directions on the GM in CVD based on bibliometric analysis of publications in this field. The findings of this study could provide a strong foundation for future research and valuable insight into the role of the GM in CVD.

## Materials and methods

### Source database and data collection

**Figure 1:**
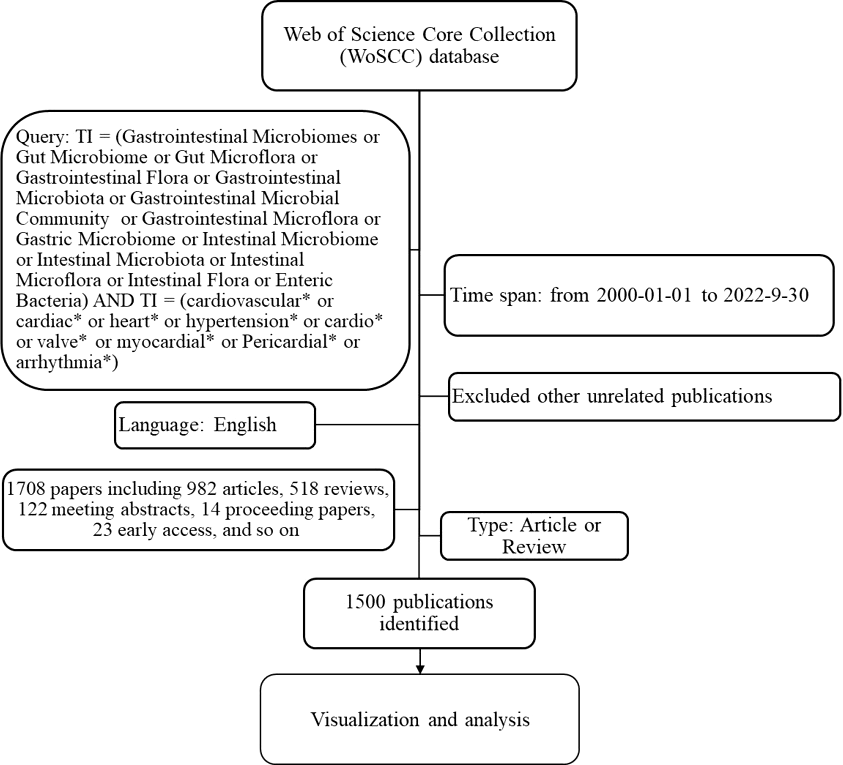
Flow chart showing the process of the selection of publications included in this study.

The source data were collected from the Web of Science™ Core Collection (WoSCC; Clarivate™, Philadelphia, PA, USA) database. The search strategy was as follows: TI (Title) = (Gastrointestinal Microbiomes or Gut Microbiome or Gut Microflora or Gastrointestinal Flora or Gastrointestinal Microbiota or Gastrointestinal Microbial Community or Gastrointestinal Microflora or Gastric Microbiome or Intestinal Microbiome or Intestinal Microbiota or Intestinal Microflora or Intestinal Flora or Enteric Bacteria) and TS (Topics) = (cardiovascular* or cardiac* or heart* or hypertension* or cardio* or valve* or myocardial* or Pericardial* or arrhythmia*). Only studies published in English were considered. A total of 1,708 publications were retrieved, including 982 articles, 518 reviews, 122 meeting abstracts, 14 proceedings papers, and 23 early-access articles. Articles or reviews were saved as “plain text”, whereas Excel files were saved as “full record and cited references”. The flow chart for the search and sorting process of the relevant articles is shown in **Figure 1**. The 2021 impact factor (IF), 2021 Journal Citation Reports (JCR), and Hirsch index (H-index) of the relevant articles were obtained directly from the WoS website.

### Data analysis and visualization

Details of publications were recorded in and analyzed using Microsoft Excel 2019. VOSviewer (version 1.6.18) (link: https://www.vosviewer.com/download) and CtieSpace (version 6.1.R3) (64-bit)] (link: https://sourceforge.net/projects/citespace/files/6.1.R3%20%28Oct%2011%2C%202022%20-%20August%2031%2C%202023%29/CiteSpace-6.1.3.msi/download) were used to process these data and to draw visual maps. VOSviewer was used to explore productive countries, authors, co-cited authors, and the most cited publications based on bibliographic data. CiteSpace was used to extract keywords from publications with high citation bursts and create the map of a timeline view of co-occurrence (keywords). The CiteSpace parameters were set as follows: time span (2004–2022), years per slice (1 year), term source (title, abstract, author keyword, keyword plus), node types (keyword), links (strength = cosine, scope = within slices), selection criteria (g-index, *k* = 25), and pruning (minimum spanning tree, pruning sliced networks, pruning the merged network). The log-likelihood rate (LLR) was adapted as the clustering algorithm, and all the clusters were labeled with keywords.

## Results

### Temporal distribution map of the literature

**Figure 2:**
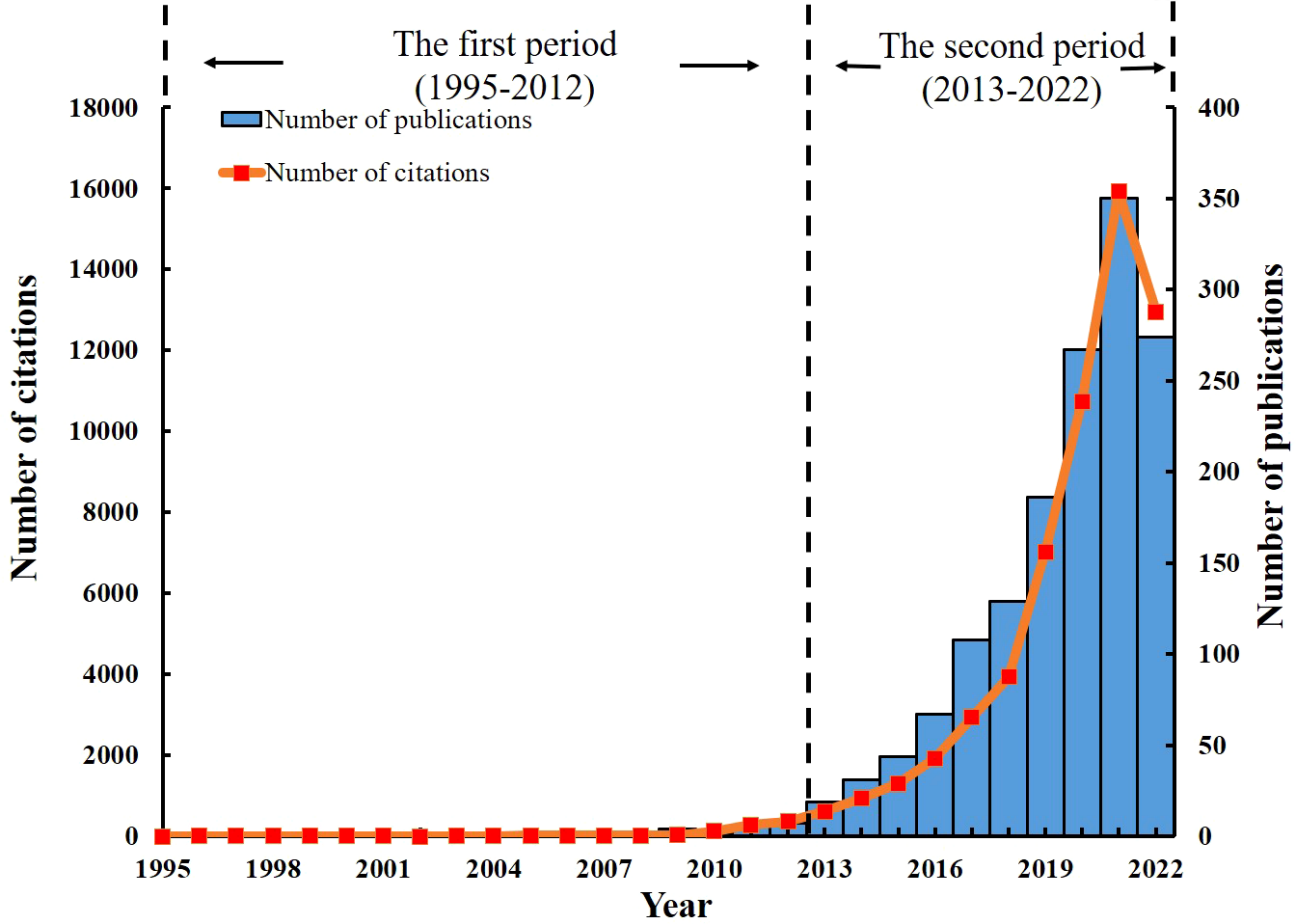
The number of annual publications and citations of articles concerning the GM in CVD from 2004 to 2022.

The search strategy yielded total of 1,708 papers, including 982 articles, 518 reviews, 122 meeting abstracts, 14 proceedings papers, and 23 early access articles. Only 1,500 articles and reviews were used in our analysis. The change in the number of publications over time reflects the development profile of a certain field. As shown in **Figure 2**, annual publications on the GM in CVD continue to increase. Two distinct phases in the development of publications can be identified. The initial growth phase took place from 2004 to 2012. The second phase began in 2013. The number of annual publications has increased sharply since then, from 19 in 2013 to 350 in 2021. Based on the number of publications in the first 9 months of 2022, the total annual number of publications this year will be more than 360. Citations also increased from 2013 (622 citations) to 2021 (15,924 citations). It is predicted that the number of citations in 2022 will be more than 170,000.

### Country distribution

**Figure 3:**
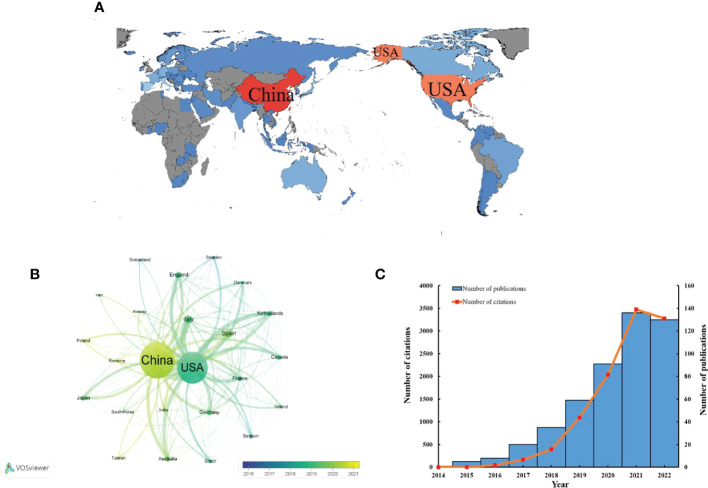
**(A)** Geographical distribution map of global publications related to the GM in CVD. **(B)** Bibliographic analysis of countries with more than 15 records over time (2016–2021). **(C)** The number of annual publications and citations of articles concerning the GM in CVD from China.

The 77 countries/regions that contributed to those 1,500 publications were included in a global map (**Figure 3A**). The colors, from blue to red, represent an increase in the number of publications. In addition, the 24 countries that contributed more than 15 publications are shown in **Figure 3B**. Each circle in the network represents a different country: the wider the circle, the more publications, and the thicker the line connecting the circles, the closer the cooperation between the countries/regions. As shown in **Figures 3A, B**, China and the USA were the most prolific countries and had the closest cooperation (**Table 1**). China (Np: 485, Nc: 9,728, H-index: 47) and the USA (Np: 418, Nc: 24,918, H-index: 82) contributed 32.31% and 27.85%, respectively, of the total number of publications, and are the two nations with the highest output of publications in this area. Surprisingly, the number of publications in this field has increased over 100-fold in the last 8 years, from only one publication in 2014 to 136 papers in 2021 (**Figure 3C**).

**Table 1:** The top 10 countries related to GM in CVD.

Rank	Country	Quantity	Nc	H-Index	ACN
1	China	485	9,728	47	20.06
2	USA	418	2,4918	82	59.61
3	Spain	95	2,623	29	27.61
4	Italy	89	3,210	30	36.07
5	England	66	3,078	29	46.64
6	Germany	61	2,504	24	41.05
7	Australia	58	2,432	24	41.93
8	Netherlands	58	2,577	21	44.43
9	Japan	57	1,991	18	34.93
10	France	54	2,252	25	41.70

NC, number of citations excluding self-citations; ACN, average number of citations.

### Analysis of authors and research institutions

**Figure 4:**
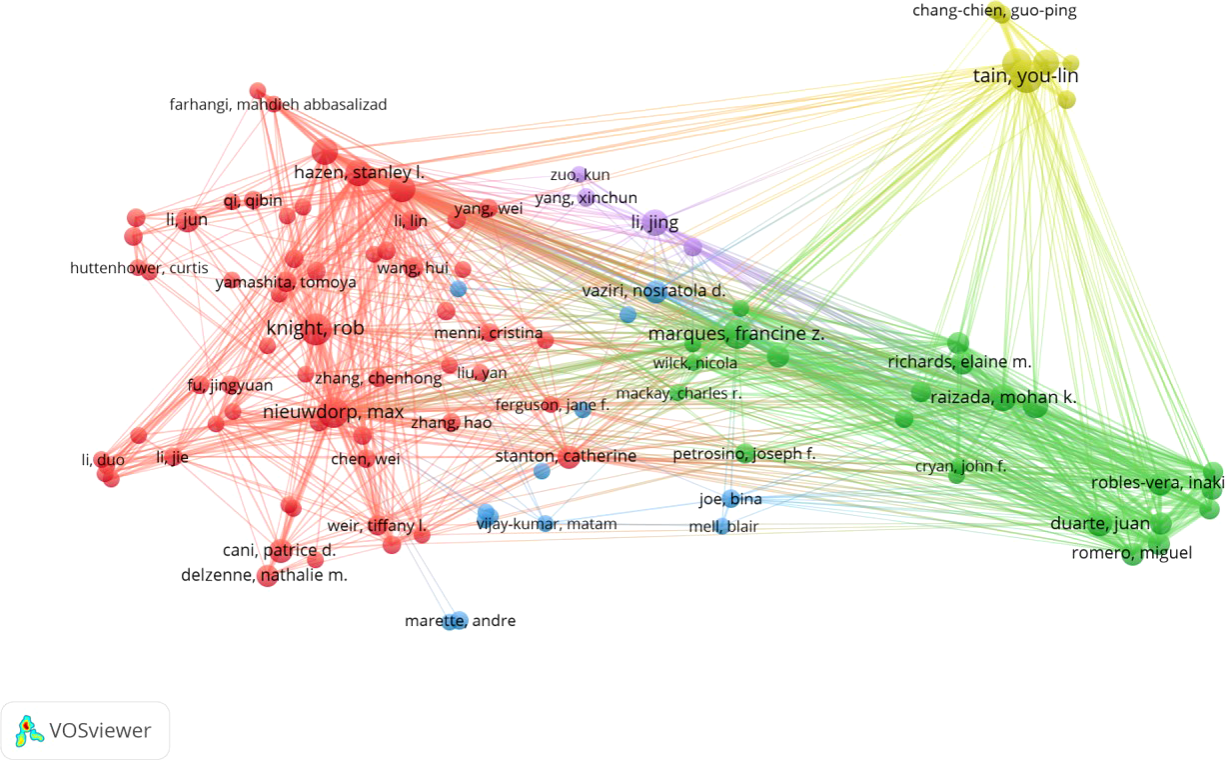
Visualization map of 103 authors with more than five papers.

A total of 8,987 authors contributed to the 1,500 publications. The top 10 most productive authors are listed in **Table 2**. Y. L. Tian (Np: 18, Nc: 262, H-index: 12) from Taiwan, China, was the most prolific author, followed by R. Knight (Np: 16, Nc: 3,036, H-index: 15) and M. Nieuwdorp (Np: 16, Nc: 503, H-index: 9). Interestingly, half of the top 10 authors were from the USA. Knight R (Nc: 3,036, ACN: 189.75), Hazen SL (Nc: 2,834, ACN: 218.0), and Tang WHW (Nc: 2,596, ACN:216.33) were the most cited authors. A total of 103 authors in this field had more than five publications. A visual analysis of the included core authors is shown in **Figure 4**. The size of the node reflects the number of publications; the different colors represent different clusters. The lines connecting different authors show that cooperation between these authors occurred.

**Table 2:** The top 10 authors of GM in CVD.

Rank	Author	Quality	Number of publications	ACN	H-index	Country
1	Tain YL	18	262	14.56	12	China
2	Knight R	16	3,036	189.75	15	USA
3	Nieuwdorp M	16	503	31.44	9	Netherlands
4	Hsu CN	14	175	12.50	10	China
5	Marques FZ	14	769	54.93	9	Australia
6	Hazen, SL	13	2,834	218.00	11	USA
7	Raizada MK	12	777	64.75	10	USA
8	Tang WHW	12	2,596	216.33	12	USA
9	Wang ZN	12	1,497	124.75	8	USA
10	Duarte J	11	221	20.09	8	Spain

ACN, average number of citations.

The top 10 institutions are listed according to the number of publications in **Table 3**. The Chinese Academy of Medical Sciences and Peking Union Medical College had the most publications (Np: 62, Nc: 3,727, H-index: 13, ACN: 60.11), followed by the University of California System (Np: 56, Nc: 3,852, H-index: 28, ACN: 68.79) in the USA and the CIBER – Centro de Investigación Biomédica en Red (Np: 46, Nc: 1,265, H-index: 19, ACN: 27.50) in Spain. Four of the top 10 institutions in this field are in China, and three are in the USA.

**Table 3:** The top 10 active institutions of GM in CVD.

Rank	Institution	Np	Nc	H-index	ACN	Country
1	Chinese Academy of Medical Sciences and Peking Union Medical College	62	3,727	13	60.11	China
2	University of California System	56	3,852	28	68.79	USA
3	CIBER – Centro de Investigación Biomédica en Red	46	1,265	19	27.50	Spain
4	Harvard University	41	2,162	18	52.73	USA
5	Udice, French Research Universities	33	1,366	18	41.39	France
6	Chinese Academy of Sciences	32	1,864	12	58.25	China
7	Institut national de la santé et de la recherche médicale (Inserm)	31	1,755	21	56.61	France
8	Sun Yat-sen University	29	303	10	10.45	China
9	Cleveland Clinic Foundation	27	3,470	19	128.52	USA
10	Shanghai Jiao Tong University	27	1,206	12	44.67	China

Np, number of publications; NC, number of citations excluding self-citations; ACN, average number of citations.

### Distribution of disciplines and journals

**Table 4** shows the top 10 disciplines in this field, in terms of the number of publications. Topping the list is “nutrition dietetics” (Np: 2 38), followed by “microbiology” (Np: 198) and “cardiovascular system cardiology” (Np: 174). Pharmacology, pharmacy, biochemistry, molecular biology, and other disciplines were also common in the literature. “Nutrition dietetics” had the highest H-index (44). The largest number of non-self-citations was for science technology and other topics (Nc: 8,437).

**Table 4:** The top 10 subject categories in the studies of in this field.

Rank	WoS category	Np	Nc	H-index
1	Nutrition Dietetics	238	5,605	44
2	Microbiology	198	8,189	41
3	Cardiovascular System Cardiology	174	7,620	42
4	Biochemistry Molecular Biology	164	4,942	37
5	Pharmacology Pharmacy	147	3,510	30
6	Food Science Technology	130	2,587	28
7	Endocrinology Metabolism	89	4,326	34
8	Research Experimental Medicine	84	4,808	28
9	Science Technology Other Topics	78	8,437	32
10	Immunology	71	2,326	21

Np, number of publications; NC, number of citations excluding self-citations.

**Table 5** shows the top 10 journals in this field. *Nutrients* had the most publications (Np: 73, Nc: 2,036, ACN: 27.89), followed by *Scientific Reports* (Np: 38, Nc: 1563, ACN: 41.13), *Frontiers in Microbiology* (Np: 34, Nc: 1,057, ACN: 31.09), and *International Journal of Molecular Sciences* (Np: 29, Nc: 740, ACN: 25.52). However, *Scientific Reports* had the highest ACN (41.132). Nine of the top 10 journals are from the JCR Q1 area. According to the most recent data (for 2021), the journal with the highest impact factor (IF) was *Nutrients* (IF: 6.706), followed by *Frontiers in Nutrition* (IF: 6.590).

**Table 5:** The top 10 journals of GM in CVD.

Rank	Journal	Np	Nc	ACN	IF (2021)	JCR (2021)
1	*Nutrients*	73	2,036	27.89	6.706	Q1
2	*Scientific Reports*	38	1,563	41.13	4.996	Q1
3	*Frontiers in Microbiology*	34	1,057	31.09	6.064	Q1
4	*International Journal of Molecular Sciences*	29	740	25.52	6.208	Q1
5	*Food & Function*	26	368	14.15	6.317	Q1
6	*Frontiers in Cellular and Infection Microbiology*	25	644	25.76	6.073	Q1
7	*Microorganisms*	25	366	14.64	4.926	Q2
8	*Frontiers in Pharmacology*	24	435	18.13	5.988	Q1
9	*Frontiers in Nutrition*	19	432	22.74	6.590	Q1
10	*PLOS One*	19	702	36.95	3.752	Q1

Np, number of publications; Nc, number of citations excluding self-citations; ACN, average number of citations.

### Highly cited literature analysis

**Figure 5:**
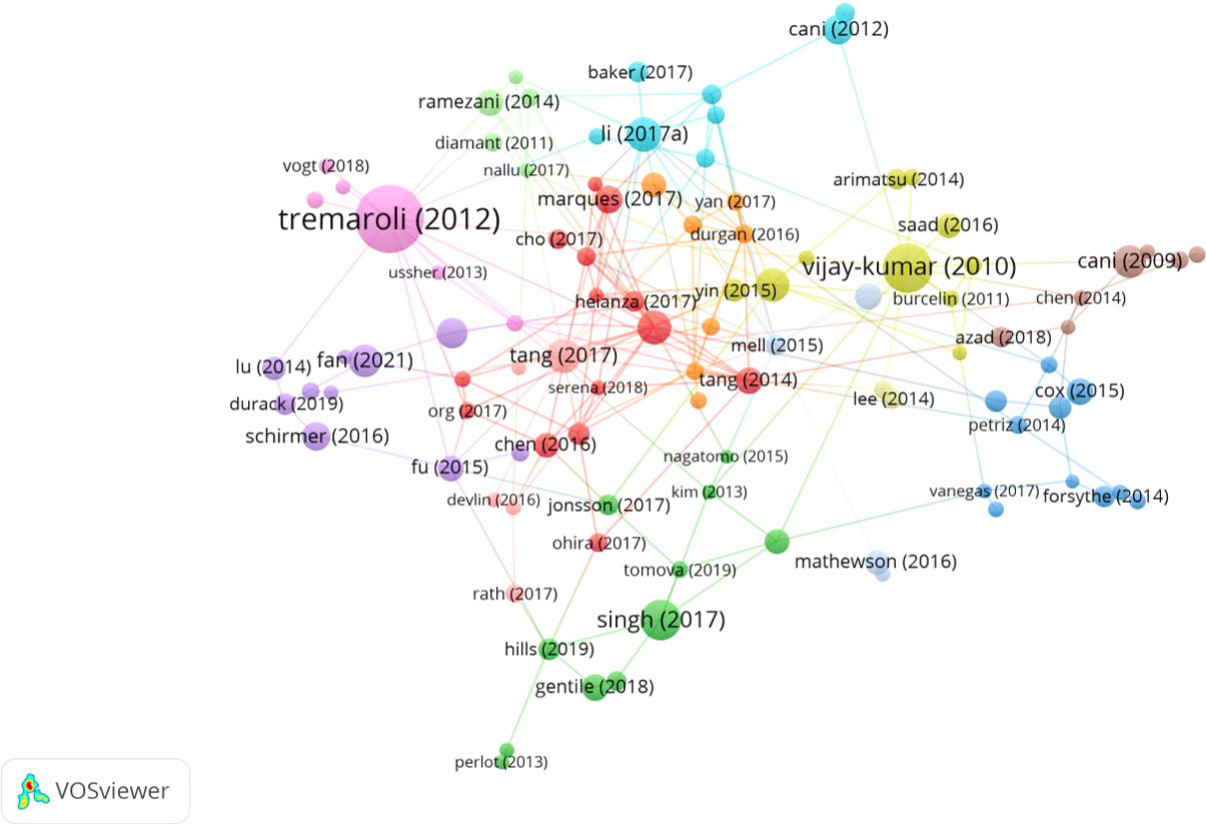
Bibliographic analysis of 115 publications with more than 120 citations.

A total of 115 papers had more than 120 citations, and these are shown in **Figure 5**, while the top 10 most cited publications are shown in **Table 6**. The size of the node corresponds to the number of citations; different colors represent different clusters. The lines between the different nodes represent cooperation between different publications. All top 10 cited publications were cited more than 500 times, and half of them were reviews. In terms of the year of publication, six of them were published after 2013.

**Table 6:** The top cited publications of GM in CVD.

Rank	Article title	Journal	Article type	Authors	Year	Number of citations
1	Functional interactions between the gut microbiota and host metabolism	*Nature*	Review	Valentina Tremaroli et al.	2012	2,621
2	Metabolic syndrome and altered gut microbiota in mice lacking Toll-like receptor 5	Science	Article	Matam Vijay-Kumar et al.	2010	1,403
3	Influence of diet on the gut microbiome and implications for human health	*Journal of Translational Medicine*	Review	Rasnik K Singh, et al.	2017	942
4	Gut microbiota in cardiovascular health and disease	*Circulation Research*	Review	W H Wilson Tang et al.	2017	680
5	Gut microbiota dysbiosis contributes to the development of hypertension	*Microbiome*	Article	Jing Li et al.	2017	670
6	Human oral, gut, and plaque microbiota in patients with atherosclerosis	*Proceedings of the National Academy of Sciences of the United States of America*	Article	Omry Koren et al.	2011	639
7	Gut microbiota-dependent trimethylamine N-oxide (TMAO) pathway contributes to both development of renal insufficiency and mortality risk in chronic kidney disease	*Circulation Research*	Article	W.H. Wilson Tang et al.	2015	635
8	The role of the gut microbiota in energy metabolism and metabolic disease	*Current Pharmaceutical Design*	Review	Patrice D Cani et al.	2009	613
9	Gut microbiota in human metabolic health and disease	*Nature Reviews Microbiology*	Review	Yong Fan et al.	2021	612
10	The gut microbiome in atherosclerotic cardiovascular disease	*Nature Communications*	Article	Zhuye Jie et al.	2017	552

The paper entitled “Functional interactions between the gut microbiota and human metabolism”, published in the journal *Nature* in 2012, with 2,621 citations, was the most cited publication. This article systematically discusses how microbes in the gut can regulate food absorption or alter host metabolic pathways through their effects on host metabolism. The review indicates that gut microbes could be the basis of new therapeutic approaches to the treatment of metabolic diseases, providing a strong foundation for further studies.

The second most cited article was “Metabolic syndrome and altered gut microbiota in mice lacking Toll-like receptor 5”, published in the journal *Science* in 2010. In this article, Matam Vijay-Kumar et al. conclude that the GM is associated with metabolic diseases and suggest that an impaired innate immune system promotes the development of metabolic diseases.

“Gut microbiota-dependent trimethylamine N-oxide (TMAO) pathway contributes to both development of renal insufficiency and mortality risk in chronic kidney disease”, with 635 citations, was the third most cited publication. The paper reports that the researchers carried out a follow-up study of 521 patients with chronic kidney disease, and found that patients with high levels of TMAO had poorer long-term survival. The authors confirmed using animal models that high levels of TMAO causes progressive renal fibrosis.

### Evolution of keywords

The keywords represent the core of the publications and can be used to determine the research frontiers of a particular field. **Table 7** shows the top 20 most mentioned keywords. Ranking first to fifth, in order, were “gut microbiota”, “cardiovascular disease”, “intestinal microbiota”, “chain fatty acids“, and “metabolism”, occurring 593, 325, 312, 240, and 194 times, respectively.

**Table 7:** Top 20 keywords in the studies of GM in CVD.

Rank	Keyword	Number of occurrences	Rank	Keyword	Number of occurrences
**1**	gut microbiota	593	**11**	diet	135
**2**	cardiovascular disease	325	**12**	disease	133
**3**	intestinal microbiota	312	**13**	gut microbiome	127
**4**	chain fatty acid	240	**14**	risk	124
**5**	metabolism	194	**15**	insulin resistance	119
**6**	trimethylamine *n*-oxide	189	**16**	metabolic syndrome	110
**7**	health	184	**17**	oxidative stress	102
**8**	inflammation	181	**18**	heart failure	101
**9**	obesity	176	**19**	bile acid	98
**10**	blood pressure	171	**20**	impact	84

**Figure 6:**
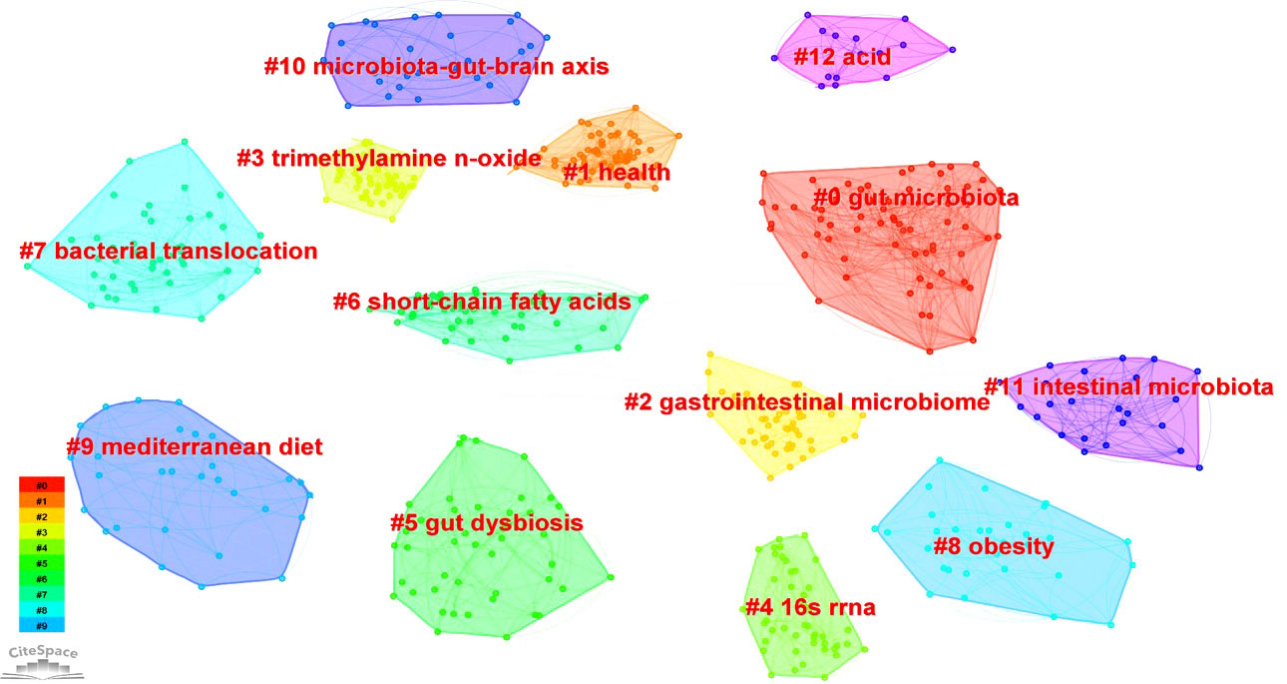
Visual analysis of top ten clusters decided by the keywords on GM in CVD performed by CiteSpace.

The top 10 clusters based on the strength of the links between keywords are shown in **Figure 6**. The cluster labels represent the primary lines of inquiry in the area, and the terms within the same cluster are highly uniform. The size of the cluster increases with an increase in the number of cluster labels. **Table 8** shows the top three most frequent keywords in each cluster. The largest grouping, cluster 0, contains 46 keywords, including “gut microbiome”, “hypertension”, and “coronary artery disease”.

**Table 8:** The main clusters of keywords in studies of the GM in CVD.

Cluster number	Label (LLR)	Size	Silhouette	Top three most frequent keywords
0	gut microbiota	71	0.726	gut microbiota; cardiovascular disease; chain fatty acid
1	health	63	0.68	health; risk; diversity
2	gastrointestinal microbiome	51	0.677	blood pressure; rat; gastrointestinal tract
3	trimethylamine *n*-oxide	50	0.687	metabolism; trimethylamine *n*-oxide; phosphatidylcholine
4	16s rRNA	46	0.632	16s rRNA; glucose; plasma
5	gut dysbiosis	45	0.766	oxidative stress; risk factor; gut dysbiosis
6	short-chain fatty acids	45	0.727	short-chain fatty acid; dietary fiber; diet-induced obesity
7	bacterial translocation	45	0.769	bacterial translocation; intestinal bacterial overgrowth; intestinal permeability
8	obesity	30	0.732	obesity; mice; diet
9	Mediterranean diet	30	0.826	Mediterranean diet; metabolite; fiber

**Figure 7:**
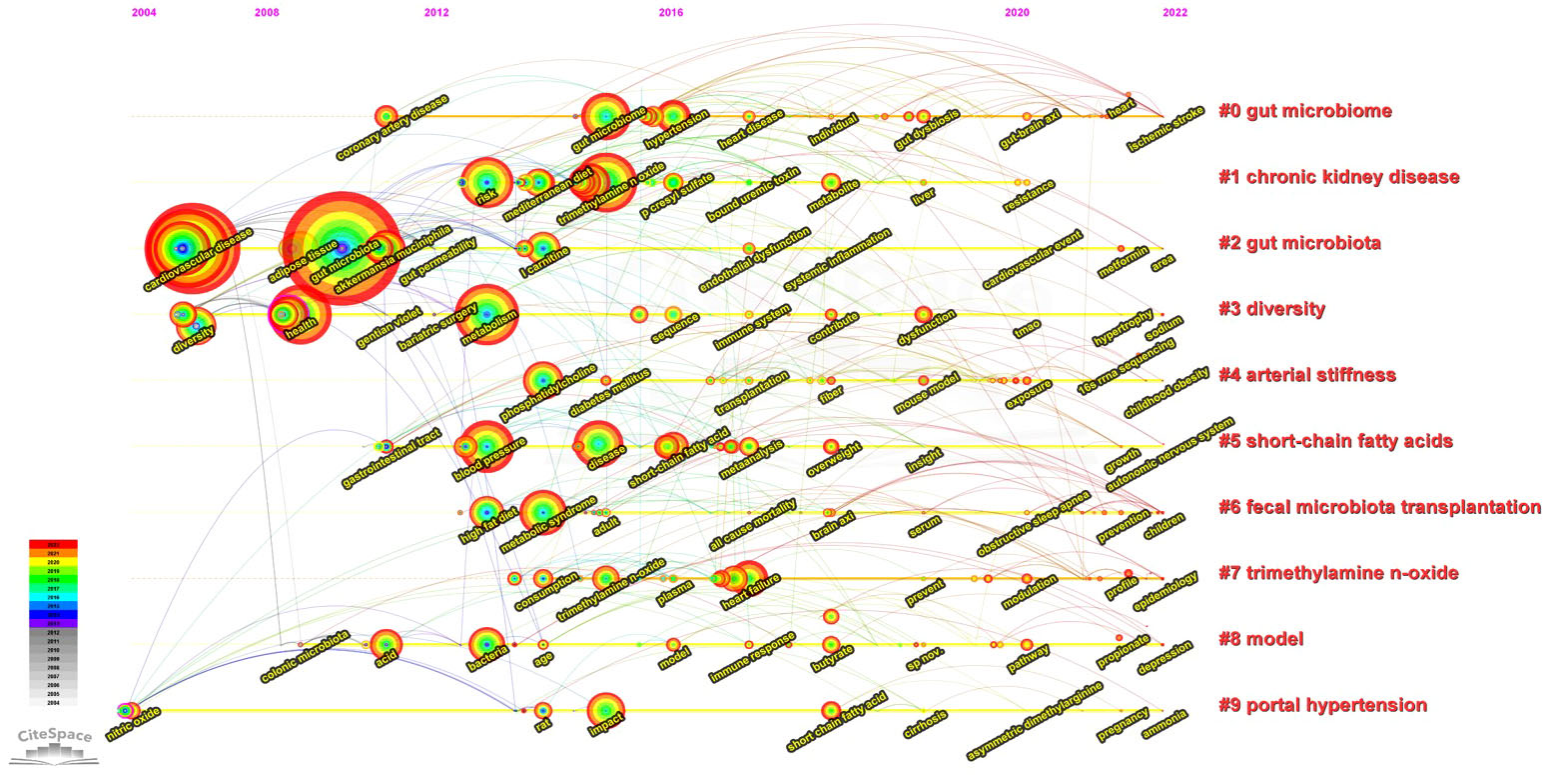
Visualization map of timeline viewer of keywords from the top ten clusters in this field by CiteSpace.

The timeline graphic of keywords in the top 10 clusters depicting the development of the high-frequency keywords is shown in **Figure 7**. In terms of the link between the GM and CVD, studies between 2004 and 2010 focused on “inflammation”, “a high-fat diet”, and “nitric oxide”. After 2010, the discipline showed increased interest in “endothelial cells”, “metabolic syndrome”, “dietary fiber”, “diabetes”, “hypertension”, and “animal model”.

### Research frontier identification

The citation burst is used to measure the innovativeness of research results, and it reflects the change of research field focus in a specific area over time. The higher the burst value, the more innovative and important the research results. To provide clues for investigating the research frontiers in the field, the top 50 keywords with the strongest citation burst intensity are shown in **Supplementary Table 1**. The blue line shows the timeline, while burst intensity is represented by a red segment on the blue timeline, which indicates the start year, end year, and duration of the burst.

The strongest burst, of 7.62 for glucagon-like peptide 1 (GLP-1), occurred in 2009, and lasted for 10 years. GLP-1 is an enteric-derived hormone stimulated by food intake. GLP-1 widely affects the function of the heart, gastrointestinal tract, liver, pancreas, brain, and adipose tissue by binding to widely distributed GLP-1 receptors. It is mainly used in the treatment of diabetes and the prevention of CVD. The longest burst duration was related to bacterial translocation (with a burst intensity of 3.61), and lasted from 2004 to 2018, highlighting the importance of the keyword.

Emerging keywords include “monooxygenase 3” (strength 3.24, 2020–2022), “short-chain fatty acid” (strength 4.63, 2021–2202), “fatty liver disease” (strength 3.18, 2021–2202), “metabolic disease” (strength 3.04, 2021–2202), “Mediterranean diet” (strength 2.95, 2021–2202), “prevention” (strength 2.77, 2021–2202), and “intestinal barrier” (strength 2.8, 2021–2202).

## Discussion

To the best of our knowledge, this is the first bibliometric analysis of the GM in CVD. Our findings highlight the following:

1. The number of publications on the GM in CVD has increased considerably in the last decade.
2. The USA and China have contributed the most in this field. The USA is more prolific than China, but China has significant potential.
3. Different research groups and countries/regions cooperate on research on the GM in CVD.
4. The journal with the most publications, among all 789 journals, was *Nutrients*.
5. Studies on the GM and “monooxygenase-3” (cluster 9), “short-chain fatty acid” (cluster 6), “fatty liver disease” (cluster 0), “Mediterranean diet” (cluster 9), “intestinal barrier” (cluster 2), and “prevention” (cluster 1) are the current hotspots and potential hotspots for future studies.

As shown in **Figure 1**, only 22 (1.53%) pieces of literature were published from 2004 to 2012, and 398 (26.5%) were published from 2013 to 2018. A total of 1,077 (71.8%) pieces of literature have been published since 2019. More than 98% of publications were published in the last decade, spurred by two novel publications by Vijay-Kumar M et al. and Omry Koren et al. In 2010, Vijay-Kumar M et al. published a paper in *Science* titled “Metabolic syndrome and altered gut microbiota in mice lacking Toll-like receptor 5”, which was the first to reveal that the GM participates in the development of metabolic diseases. The research also indicated that dysfunction of the innate immune system promotes the development of metabolic syndrome (Vijay-Kumar et al., 2010). In early 2011, Omry Koren et al. published another paper, titled “Human oral, gut, and plaque microbiota in patients with atherosclerosis”, in *Proceedings of the National Academy of Sciences of the USA.* Their study showed that bacteria from the oral cavity and the gut correlate with disease markers of atherosclerosis (Koren et al., 2011). Those two papers set the pace and provided a strong foundation for research on the role of the GM in CVD. Two reviews, “The role of the gut microbiota in energy metabolism and metabolic disease” by Cani PD et al., published in *Current Pharmaceutical Design* in 2009 (Cani and Delzenne, 2009), and “Functional interactions between the gut microbiota and host metabolism”, by Tremaroli V et al., published in *Nature* in 2012 (Tremaroli and Backhed, 2012), systematically summarized the research in this field, and showed research prospects in this area. Notably, those four papers were among the top 10 most cited papers, and were published before 2013, before a rapid increase in publications in this field.

The USA and China contributed similar numbers of publications in this field, 418 (27.8%) and 485 (32.3%) publications, respectively. This indicator is highly dependent on the number of productive institutions in a country. The USA and China are leading countries in publications in this field, with three and four institutions, respectively, among the top 10 institutions with the most publications in this field. As shown in **Table 3**, the Chinese Academy of Medical Sciences and Peking Union Medical College (Np: 62), the Chinese Academy of Sciences (Np: 33), Sun Yat-sen University (Np: 29), and Shanghai Jiao Tong University (Np: 27) were the four Chinese institutions in the top 10 most productive institutions in this field. The University of California System (Np: 56), Harvard University (Np: 41), and the Cleveland Clinic Foundation (Np: 27) were the three USA institutions in the top 10 fruitful institutions. China had seven of the top 10 most prolific authors, which explains its position among the top 10 fruitful countries. Furthermore, differences in the influence of the USA and China should be noted. The H-index (47) and ACN (20.1) were lower for China than for the USA (H-index: 80, ACN: 59.6). As shown in **Figure 3C**, China began research in this field much later than the USA, which might explain why it lags the USA. As in other fields of cardiology (Lai et al., 2017), the USA’s excellence in the scientific research system offered a strong base for high-quality research. Knight R, who is from the USA, has cooperated widely with other authors, which further underlines the great influence of the USA in this area of study (**Figure 4**). The huge variety of the GM and the complexity of the mechanisms linking the GM and CVD make research in this field difficult (Rahman et al., 2022). Cooperation between different groups or institutions is necessary if the hidden mechanisms are to be determined. The number of publications from China has increased hugely in the last 8 years, from 1 in 2014 to 136 in 2021. In the first 9 months of 2022, the number of publications from China was 130, and the total number of publications in 2022 will certainly be higher than in 2021. This suggests that China will become more influential in this field in the future.

The papers in this field were published in 789 journals. The journal *Nutrients* accounted for the largest number of publications (73), followed by other journals such as *Scientific Reports* (Np: 38), *Frontiers in Microbiology* (Np: 34), and *Food & Function* (Np: 26). *Nutrients* publishes articles on a wide range of topics, including diet-related disorders, metabolic syndrome, public health, and so on. The GM plays an important role in nutrient supply and balance (Valdes et al., 2018) but also participates in the pathological process of different diseases (Martinez et al., 2009). Therefore, studies on the GM and CVD fit well with the scope of *Nutrients*. Recently, more mechanisms of how the GM leads to disease have been discovered, and more than 10 papers related to the GM and CVD have been published in *Nutrients* in last 2 years (Liu et al., 2022; Burakova et al., 2022). It can be expected that, in the future, more papers on interesting topics in this field will published in this journal.

All keywords, clusters, and the top 20 keywords with the strongest citation bursts are shown in **Figures 6**, **7**, **Table 7**, and **Supplementary Table 1**. Keywords represent the core content of a study. The evolution of keywords reflects the research direction of studies in the field. A cluster contains related keywords on a specific theme. In this field, all keywords were divided into 10 main clusters, with each cluster indicating a research direction. Cluster 8 (obesity) includes all studies related to obesity and GM. Obesity is one of the leading risk factors for CVD. Cluster 6 (short-chain fatty acids) comprises studies on the relationship between the GM and short-chain fatty acids. Short-chain fatty acids mediate the progress of CVD (Nogal et al., 2021) and have been implicated in the pathophysiology of atherosclerosis (Ohira et al., 2017). Apart from classifying keywords by cluster, the evolutionary path in different clusters revealed additional details in each cluster, reflecting the evolution of keywords over time. The keyword most representative of cluster 0 was initially, i.e., in 2010, “high-fat diet” but became “metabolic syndrome” in 2015 and “fatty liver disease” and “epithelial cell” in recent times. In cluster 1, the main keyword evolved from “risk” in around 2012 to “marker” in 2018, and to “prevention”, and “modulation” in recent times. “Atherosclerosis” was the most common keyword in cluster 3 in 2010, and has evolved to “metformin nutrients” in recent years. “Gut microbiota dysbiosis” was the earliest keyword and “metabolic disease” and “epidemiology” are the latest keywords in cluster 4. The earliest keyword in cluster 7 was “nitric oxide” in 2005, while recent keywords in this cluster are “pulse wave velocity” and “ammonia”. “Bacteria” was initially, in 1995, the most common keyword in cluster 8, but “inflammation” is now the most popular keyword in this cluster. “Childhood obesity” is the latest keyword in this cluster. In cluster 9, “carnitine” was the most common keyword in 2016, but has been overtaken by “cardiometabolic disease”. The emerging keywords in a specific cluster indicate the research trend or the potential research hotspot in that field.

Keywords with strong citation bursts indicate the emerging research direction. Monooxygenase-3 (cluster 9) participates in cholesterol metabolism (Schugar and Brown, 2015). It has now been confirmed to be associated with dioxin-induced reorganization of the gut microbiome and host insulin sensitivity (Massey et al., 2022). Short-chain fatty acids (cluster 6) were shown in early 2006 to improve colonic and systemic health (Wong et al., 2006), but, with the development of GM studies, the function of short-chain fatty acids has been further elucidated (Chambers et al., 2018; Bartolomaeus et al., 2019), and it is likely that short-chain fatty acids will continue to be a hot topic in the future. The cause of fatty liver disease (cluster 0), especially non-alcoholic fatty liver disease, is the subject of debate (Chiriac et al., 2021), but, interestingly, it is now thought that the GM may be involved (Drozdz et al., 2021). A Mediterranean diet (cluster 9) has been confirmed to be beneficial for CVD and some chronic diseases. However, the detailed mechanisms should be explored in randomized controlled trials (Merra et al., 2020). Intestinal barrier (cluster 2) dysfunction is associated with CVD (Li et al., 2020), and, thus, regulation of intestinal barrier function has been suggested as a promising novel therapeutic target for treating CVD (Lewis and Taylor, 2020). As the role of the GM in CVD is complicated, targeting the GM may be a double-edged sword. Despite leading to CVD, the GM is also a target for preventing CVD (Yamashita et al., 2015; Hsu et al., 2021), which is why “prevention” (cluster 1) is an emerging keyword. Although some of the emerging keywords are not immediately associated with GM and CVD, they indicate the current research direction in this field. Generally, these keywords show potential hotspots.

### Limitations

Despite our best efforts to include as many data as possible and to ensure that this study was fair and reliable, certain limitations could not be avoided. First, we examined only publications from the WoS database. The results may have been different if we had used other databases. Second, only studies published in English were considered. Thus, some important studies published in other languages may have been omitted. Third, the results in this study were analyzed based on author keywords and keywords plus, but the results may have been different if index keywords or abstract keywords had been used.

## Conclusions

Research on the role of the GM in CVD has become a hotspot area in recent years. Publications on the GM in CVD have increased rapidly in the last 10 years. China is the leading country in the field, with the highest number of publications on the GM in CVD, with the USA coming a close second. However, the USA is the country that is most influential in this field. Close cooperation between different authors and countries promotes progress in this field. The journal *Nutrients*, among a total of 789 journals, has the highest number of publications in this field, and studies on “monooxygenase-3”, “short-chain fatty acid”, “fatty liver disease”, “metabolic disease”, “Mediterranean diet”, “intestinal barrier”, and “prevention” are likely to be published in this field. The findings of this study provided valuable insights into future studies on GM in CVD.

## Data availability statement

The original contributions presented in the study are included in the article/**Supplementary Material**. Further inquiries can be directed to the corresponding authors.

## Author contributions

PL, Y-lL, and MS designed the study. MS, W-wC, and SqX collected and chose the data. PL, MS, SqX, W-wC, F-qL, Y-xO, and Y-mZ analyzed the data and drew the figures; PL, SqX, and MS drafted the manuscript. PL and Y-lL revised the final version of the manuscript. The authors read and approved the final manuscript.
