# Supplementary material for: A bibliometric analysis of studies on the gut microbiota in cardiovascular disease from 2004 to 2022
Source: Front Cell Infect Microbiol. 2023 Jan 6;12:1083995. doi: 10.3389/fcimb.2022.1083995 (PMC9852829; doi:10.3389/fcimb.2022.1083995)
Supplement: Supplementary file 1 [file Table_1.docx]

Supplementary Table 1. Top 50 keywords with the strongest citation bursts.

| **Keywords** | **Strength** | **Begin** | **End** | **2004-2022** |
| --- | --- | --- | --- | --- |
| bacterial translocation | 3.61 | 2004 | 2018 | ▃▃▃▃▃▃▃▃▃▃▃▃▃▃▃▂▂▂▂ |
| 16s ribosomal rna | 4.37 | 2006 | 2015 | ▂▂▃▃▃▃▃▃▃▃▃▃▂▂▂▂▂▂▂ |
| glucagon like peptide 1 | 7.62 | 2009 | 2018 | ▂▂▂▂▂▃▃▃▃▃▃▃▃▃▃▂▂▂▂ |
| adipose tissue | 6.93 | 2009 | 2018 | ▂▂▂▂▂▃▃▃▃▃▃▃▃▃▃▂▂▂▂ |
| high fat diet | 5.65 | 2009 | 2016 | ▂▂▂▂▂▃▃▃▃▃▃▃▃▂▂▂▂▂▂ |
| community | 4.85 | 2009 | 2019 | ▂▂▂▂▂▃▃▃▃▃▃▃▃▃▃▃▂▂▂ |
| diet induced obesity | 7.41 | 2010 | 2016 | ▂▂▂▂▂▂▃▃▃▃▃▃▃▂▂▂▂▂▂ |
| mice | 4.97 | 2010 | 2016 | ▂▂▂▂▂▂▃▃▃▃▃▃▃▂▂▂▂▂▂ |
| coronary heart disease | 3.08 | 2010 | 2016 | ▂▂▂▂▂▂▃▃▃▃▃▃▃▂▂▂▂▂▂ |
| gastrointestinal tract | 3.27 | 2011 | 2016 | ▂▂▂▂▂▂▂▃▃▃▃▃▃▂▂▂▂▂▂ |
| weight lo | 4.27 | 2012 | 2016 | ▂▂▂▂▂▂▂▂▃▃▃▃▃▂▂▂▂▂▂ |
| c reactive protein | 4.25 | 2013 | 2015 | ▂▂▂▂▂▂▂▂▂▃▃▃▂▂▂▂▂▂▂ |
| l carnitine | 6.17 | 2014 | 2017 | ▂▂▂▂▂▂▂▂▂▂▃▃▃▃▂▂▂▂▂ |
| indoxyl sulfate | 4.87 | 2014 | 2018 | ▂▂▂▂▂▂▂▂▂▂▃▃▃▃▃▂▂▂▂ |
| phosphatidylcholine | 3.66 | 2014 | 2017 | ▂▂▂▂▂▂▂▂▂▂▃▃▃▃▂▂▂▂▂ |
| activated protein kinase | 2.91 | 2014 | 2017 | ▂▂▂▂▂▂▂▂▂▂▃▃▃▃▂▂▂▂▂ |
| intestinal bacterial overgrowth | 2.69 | 2014 | 2017 | ▂▂▂▂▂▂▂▂▂▂▃▃▃▃▂▂▂▂▂ |
| rat | 4.78 | 2015 | 2016 | ▂▂▂▂▂▂▂▂▂▂▂▃▃▂▂▂▂▂▂ |
| metabolism | 4.06 | 2015 | 2015 | ▂▂▂▂▂▂▂▂▂▂▂▃▂▂▂▂▂▂▂ |
| dietary fiber | 3.69 | 2015 | 2017 | ▂▂▂▂▂▂▂▂▂▂▂▃▃▃▂▂▂▂▂ |
| absorption | 2.67 | 2015 | 2016 | ▂▂▂▂▂▂▂▂▂▂▂▃▃▂▂▂▂▂▂ |
| intestinal microbiota | 4.28 | 2016 | 2016 | ▂▂▂▂▂▂▂▂▂▂▂▂▃▂▂▂▂▂▂ |
| hemodialysis patient | 4.26 | 2016 | 2019 | ▂▂▂▂▂▂▂▂▂▂▂▂▃▃▃▃▂▂▂ |
| in vivo | 3.50 | 2016 | 2017 | ▂▂▂▂▂▂▂▂▂▂▂▂▃▃▂▂▂▂▂ |
| physical activity | 3.38 | 2016 | 2017 | ▂▂▂▂▂▂▂▂▂▂▂▂▃▃▂▂▂▂▂ |
| inflammatory bowel disease | 3.32 | 2016 | 2017 | ▂▂▂▂▂▂▂▂▂▂▂▂▃▃▂▂▂▂▂ |
| chronic kidney disease | 3.10 | 2016 | 2018 | ▂▂▂▂▂▂▂▂▂▂▂▂▃▃▃▂▂▂▂ |
| p cresyl sulfate | 3.04 | 2016 | 2018 | ▂▂▂▂▂▂▂▂▂▂▂▂▃▃▃▂▂▂▂ |
| kidney disease | 2.90 | 2016 | 2017 | ▂▂▂▂▂▂▂▂▂▂▂▂▃▃▂▂▂▂▂ |
| glucose | 2.76 | 2016 | 2018 | ▂▂▂▂▂▂▂▂▂▂▂▂▃▃▃▂▂▂▂ |
| chronic renal failure | 4.74 | 2017 | 2019 | ▂▂▂▂▂▂▂▂▂▂▂▂▂▃▃▃▂▂▂ |
| fecal microbiota | 3.86 | 2017 | 2018 | ▂▂▂▂▂▂▂▂▂▂▂▂▂▃▃▂▂▂▂ |
| bound uremic toxin | 3.16 | 2017 | 2019 | ▂▂▂▂▂▂▂▂▂▂▂▂▂▃▃▃▂▂▂ |
| intestinal microbiota | 2.99 | 2017 | 2020 | ▂▂▂▂▂▂▂▂▂▂▂▂▂▃▃▃▃▂▂ |
| atherosclerosis | 2.74 | 2017 | 2017 | ▂▂▂▂▂▂▂▂▂▂▂▂▂▃▂▂▂▂▂ |
| arterial stiffness | 2.83 | 2018 | 2019 | ▂▂▂▂▂▂▂▂▂▂▂▂▂▂▃▃▂▂▂ |
| therapy | 2.81 | 2018 | 2019 | ▂▂▂▂▂▂▂▂▂▂▂▂▂▂▃▃▂▂▂ |
| polyphenol | 3.31 | 2019 | 2019 | ▂▂▂▂▂▂▂▂▂▂▂▂▂▂▂▃▂▂▂ |
| human health | 3.12 | 2019 | 2020 | ▂▂▂▂▂▂▂▂▂▂▂▂▂▂▂▃▃▂▂ |
| mouse model | 3.01 | 2019 | 2019 | ▂▂▂▂▂▂▂▂▂▂▂▂▂▂▂▃▂▂▂ |
| intervention | 2.81 | 2019 | 2019 | ▂▂▂▂▂▂▂▂▂▂▂▂▂▂▂▃▂▂▂ |
| cirrhosis | 2.73 | 2019 | 2020 | ▂▂▂▂▂▂▂▂▂▂▂▂▂▂▂▃▃▂▂ |
| pathway | 3.33 | 2020 | 2020 | ▂▂▂▂▂▂▂▂▂▂▂▂▂▂▂▂▃▂▂ |
| monooxygenase 3 | 3.24 | 2020 | 2022 | ▂▂▂▂▂▂▂▂▂▂▂▂▂▂▂▂▃▃▃ |
| short-chain fatty acid | 4.63 | 2021 | 2022 | ▂▂▂▂▂▂▂▂▂▂▂▂▂▂▂▂▂▃▃ |
| fatty liver disease | 3.18 | 2021 | 2022 | ▂▂▂▂▂▂▂▂▂▂▂▂▂▂▂▂▂▃▃ |
| metabolic disease | 3.04 | 2021 | 2022 | ▂▂▂▂▂▂▂▂▂▂▂▂▂▂▂▂▂▃▃ |
| mediterranean diet | 2.95 | 2021 | 2022 | ▂▂▂▂▂▂▂▂▂▂▂▂▂▂▂▂▂▃▃ |
| intestinal barrier | 2.8 | 2021 | 2022 | ▂▂▂▂▂▂▂▂▂▂▂▂▂▂▂▂▂▃▃ |
| prevention | 2.77 | 2021 | 2022 | ▂▂▂▂▂▂▂▂▂▂▂▂▂▂▂▂▂▃▃ |
